# *Tsukamurella tyrosinosolvens* Respiratory Infection in Immunocompetent Man

**DOI:** 10.3201/eid3103.241365

**Published:** 2025-03

**Authors:** Aidan Clifford, Jenny Siaw Jin Wong, Ben Aw-Yeong, Kerrie Lea, Maria Globan, Benjamin Smith

**Affiliations:** Western Health, Melbourne, Victoria, Australia (A. Clifford, B. Smith); Dorevitch Pathology, Melbourne (J. Siaw Jin Wong, B. Aw-Yeong); Victorian Infectious Disease Reference Laboratory at the Peter Doherty Institute for Infection and Immunity, Melbourne (K. Lea, M. Globan)

**Keywords:** *Tsukamurella tyrosinosolvens*, bacteria, bacterial infection, respiratory diseases, Australia, India

## Abstract

*Tsukamurella* spp. are an infrequent and underdiagnosed cause of bacterial respiratory infection, usually occurring in patients with structural lung disease or immune compromise. We describe *T. tyrosinosolvens* respiratory infection in a patient in Australia without structural lung disease or known immune deficiency. The patient was successfully treated with oral ciprofloxacin and clarithromycin.

*Tsukamurella* spp. are variably or weakly acid-fast, gram-positive, non–spore-forming, obligate aerobic actinomycetes and are typically isolated from water, soil, and other terrestrial samples. *Tsukamurella* infections are rare but, when reported, are often associated with indwelling vascular or peritoneal catheters (*1*). *Tsukamurella* spp. also have been described as causative agents in respiratory infection, meningitis, keratitis, cutaneous infection, and acute otitis media (*1*,*2*). Nonpathogenic respiratory colonization by *Tsukamurella* spp. also has been described (*1*). *Tsukamurella* respiratory infection probably is underidentified because of clinical, radiologic, and morphologic similarities with related, more common organisms such as *Mycobacterium tuberculosis*. The advent of DNA and RNA sequencing techniques and matrix-assisted laser desorption/ionization time-of-flight (MALDI-TOF) mass spectrometry has enabled an increase in diagnosis of *Tsukamurella* infection (*2*,*3*).

*Tsukamurella* spp. respiratory infection might be clinically indistinguishable from pulmonary tuberculosis (TB); symptoms include cough, hemoptysis, and weight loss (*1*). Current understanding of risk factors is limited; however, 38% of previously described cases were associated with immune compromise and 69% with underlying structural lung disease (*1*,*4*–*13*) (Appendix). *Tsukamurella* pulmonary co-infection with other aerobic actinomycetes also has been described (*13*).

Because reported cases are rare, awareness of *Tsukamurella* spp. infection among clinicians is limited, and evidence to guide empiric management is scarce. We describe a case of *T. tyrosinosolvens* respiratory infection in an apparently immunocompetent patient in Australia without underlying structural lung disease.

## The Case-Patient

A 25-year-old man, 3 years postmigration from India, was referred to our hospital with right apical nodular opacities on chest radiograph performed during routine migrant screening, which were presumed to be attributable to TB. He was asymptomatic, with no cough, dyspnea, fevers, night sweats, or recent weight loss. The patient had no remarkable medical history and did not take medications regularly. He reported no smoking or recreational drug use. He had no history of immune deficits such as HIV infection, malignancy, stem cell or solid organ transplant, steroid or other immunosuppressive drug use, diabetes mellitus, alcoholism, renal failure, liver failure, or previous splenectomy. He worked in manufacturing and had exposure to diesel engines and reported no exposure to soil, animals, or wastewater.

At initial assessment, the patient was afebrile and had no focal respiratory signs. Initial blood testing results were unremarkable; C-reactive protein and leukocyte counts were within reference ranges. Results of assay tests for TB and HIV were negative. Sputum specimens collected on 3 consecutive days were smear-negative for acid-fast bacilli (AFB) with auramine-rhodamine stain.

Computed tomography (CT) of the chest showed variable-sized pulmonary nodules in the right lung apex and surrounding tree-in-bud changes (Figure). CT also identified 2 cavities; the larger cavity measured 25 mm and contained an air-fluid level. The remaining lung was clear. Cultures of all 3 sputum samples indicated *Tsukamurella* spp. most closely related to *T. tyrosinosolvens*. We performed species identification through targeted Sanger DNA sequencing of a 550-bp fragment of the 16S rRNA gene, followed by BLAST analysis (https://blast.ncbi.nlm.nih.gov) to determine the most closely matched species. Given radiologic findings consistent with active infection and repeated isolation of *Tsukamurella* spp., we decided to treat empirically with ciprofloxacin and clarithromycin (both 500 mg 2/d).

**Figure:**
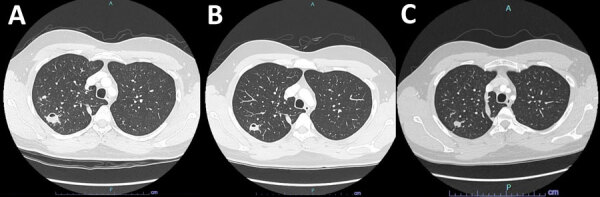
Computed tomography (CT) scans for reported case of *Tsukamurella tyrosinosolvens* infection in 25-year-old immunocompetent man. A) Initial CT showing 25-mm cavitating right apical lesion. B) Repeat CT after 6 months of treatment, indicating interval cavitary lesion reduction to 10 mm and resolution of the second smaller cavitation seen on original CT. C) Final CT 6 months after treatment cessation, indicating further cavity resolution without evidence of infection recurrence.

Subsequent sensitivity testing through broth microdilution demonstrated susceptibility to a broad range of antimicrobial drugs, intermediate susceptibility to doxycycline, and resistance to amoxicillin/clavulanic acid and tobramycin (Table). Given the lack of clinical data to support development of validated MIC breakpoints for *Tsukamurella* spp*.,* we interpreted susceptibilities by using MIC breakpoints for *Nocardia* spp. by Clinical and Laboratory Standards Institute guidelines (*14*). Two further sputum samples collected 1 month later also were AFB smear-negative; however, we cultured *Mycobacterium fortuitum* from a single specimen, which was not considered causative or otherwise clinically important.

**Table:** Antimicrobial susceptibility testing results for reported case of *Tsukamurella tyrosinosolvens* infection in 25-year-old immunocompetent man, with tentative interpretation based on *Nocardia* spp. breakpoints (*14*)*

Agent	MIC, μg/mL	Tentative interpretation
Amikacin	1	S
Amoxicillin/clavulanic acid	64/32	R
Ceftriaxone	4	S
Ciprofloxacin	0.5	S
Clarithromycin	2	S
Doxycycline	4	I
Imipenem	2	S
Linezolid	2	S
Minocycline	1	S
Moxifloxacin	0.25	S
Tobramycin	16	R
Trimethoprim/sulfamethoxazole	0.25/4.75	S

*I, intermediate; R, resistant; S, sensitive.

After 3 months of treatment, the patient remained asymptomatic and reported no adverse events associated with treatment. Repeat CT showed interval reduction of the cavitating lesion from 25 to 17 mm (Figure). Treatment was continued for another 3 months, at which time the nodules had further reduced in size; the cavitating lesion measured 10 mm (Figure). After 6 months of macrolide and fluoroquinolone therapy, treatment was stopped. We noted no radiologic signs of infection recurrence on repeat CT at 6 months after treatment cessation.

## Conclusions

We describe a case of *T. tyrosinosolvens* infection as a cause of cavitating respiratory disease in an immunocompetent and otherwise healthy young man. This case challenges the characterization of *Tsukamurella* spp. as opportunistic pathogens and should raise awareness of *Tsukamurella* respiratory infection. Although *Tsukamurella* pulmonary infection is rare, the number of reports is increasing, and most cases have been published within the last decade (Appendix). The emergence of *Tsukamurella* bacteria as a cause of human infection probably reflects advances in laboratory methods and increased recognition of a previously misdiagnosed disease. Although the true prevalence remains unclear, epidemiologic studies in China indicated that 1% of presumed nontuberculous *Mycobacteria* respiratory samples were *Tsukamurella* spp. when they were retrospectively analyzed using molecular methods (*15*).

Underrecognition occurs for several reasons. Lack of awareness among clinicians and the practice of treating AFB culture-positive infection as presumed TB in some clinical settings contribute to misdiagnosis (*2*,*7*). Because *Tsukamurella* spp. respiratory infection might be clinically, radiologically, and morphologically indistinguishable from pulmonary TB and might also respond to treatment with first-line TB therapy, the risk for misdiagnosis is high in the absence of microbiologic confirmation. A broader differential including other aerobic actinomycetes could be beneficial, especially in patients not responding to initial therapy. *Tsukamurella* resistance to first-line TB treatment agents has been described (*2*). Misdiagnosis has been shown to lead to excess disease and death in some case reports (*7*) and can also lead to use of unnecessary, toxicity-prone TB treatment regimens.

Microbiologic diagnosis often is resource-intensive. Standard laboratory phenotypic and biochemical methods might be inadequate to distinguish *Tsukamurella* from other aerobic actinomycetes. The advent of MALDI-TOF mass spectrometry and molecular techniques such as 16S rRNA and DNA sequencing have enabled accurate identification of *Tsukamurella* genus (*2*). Although 16S sequencing is an effective technique for identifying *Tsukamurella* genus, it often is insufficient to achieve species-level identification because of the high genetic conservation between species and lack of sequences available on public databases for comparison. Sequencing of additional housekeeping genes (e.g., *groEL*, *sec*A, and *rpoB*) might be necessary for *Tsukamurella* species identification. Although some literature describes low diagnostic accuracy of *Tsukamurella* spp. with MALDI-TOF mass spectrometry, recent database improvements have demonstrated species-level identification with 98% accuracy (*3*). Therefore MALDI-TOF mass spectrometry might be an efficacious and cost-effective diagnostic method compared with DNA and RNA sequencing techniques. Unfortunately, those methods might not be routinely available outside metropolitan clinical microbiology and reference laboratories and can be impractical in settings with high TB prevalence.

Even once diagnosis is made, evidence to guide antibiotic choice and duration of therapy is scarce. Empiric regimens are based on previous case reports, and duration must be guided by clinical response. In the absence of validated MIC breakpoints, correlation between in vivo and in vitro sensitivities might be poor. Current Clinical and Laboratory Standards Institute guidelines provide tentative breakpoints for interpreting *Tsukamurella* spp. susceptibility testing on the basis of *Nocardia* spp. breakpoints (*14*); however, patients should be monitored to ensure appropriate clinical response. 

Appendix Additional information about *Tsukamurella tyrosinosolvens* respiratory infection in immunocompetent man.
