# Supplementary material for: Tsukamurella tyrosinosolvens Respiratory Infection in Immunocompetent Man
Source: Emerg Infect Dis. 2025 Mar;31(3):596–9. doi: 10.3201/eid3103.241365 (PMC11878327; doi:10.3201/eid3103.241365)
Supplement: Appendix — Additional information about Tsukamurella tyrosinosolvens respiratory infection in immunocompetent man. [file 24-1365-Techapp-s1.pdf]

Article DOI: https://doi.org/10.3201/eid3103.241365

EID cannot ensure accessibility for supplementary materials supplied by authors. Readers who have difficulty accessing supplementary content should contact the authors for assistance.

Tsukamurella tyrosinosolvens Respiratory Infection in Immunocompetent Man

Appendix

Appendix Table. Summary table of previously described *Tsumakurella sp.* respiratory tract infections

First author (year)	Details	Risk factors	Clinical presentation	Radiology	Diagnostic method and species	Treatment
Menard et al, 2009 (*1*)	54M with Tsukamurella bronchitis and bacteremia	Lung transplant, α-1 antitrypsin deficiency	Fever and productive cough for 2 weeks	N/A	*T. tyrosinosolvens* confirmed using 16S and hsp65 sequencing	Imipenem and tobramycin
Maalouf et al, 2009 (*2*)	74M with Tsukamurella pneumonia	COPD and Non-Hodgkins Lymphoma	4 d of fever, cough and dyspnoea	CXR: Bilateral perihilar alveolar infiltrates	*Tsukamurella* pulmonis confirmed using 16S	Vancomycin and meropenem. Switched to 10 d oral ciprofloxacin and rifampin.
Perez et al, 2008 (*3*)	71M with cavitating lung lesion	Lung adenocarcinoma	3 mo of productive cough, hemoptysis, fevers, night sweats	CT: LUL cavity	*Tsukamurella* pulmonis diagnosed with biochemical methods	Rifampin and levofloxacin for 6 mo.
Alcaide et al, 2004 (*4*)	55M with cavitary pneumonia	AIDS, T-cell lymphoma and COPD	2 weeks of cough, fever, malaise.	CT: multiple cavities with patchy areas of consolidation.	*Tsukamurella* genus diagnosed with biochemical methods.	Oral ciprofloxacin and rifabutin for 12 weeks.
Mehta et all, 2011 (*5*)	79M with strep pneumoniae and Tsukamurella pneumonia	Emphysema	10 d of productive cough and fever	CT: Right upper lobe dense infiltration.	*Tsukamurella sp.* diagnosed with chromatography	Started HRZE. Changed to ciprofloxacin and azithromycin for 2 mo.
Inchingolo et al, 2010 (*6*)	76F with polymicrobial pneumonia	COPD on home O2	2 of days confusion and dyspnoea	CXR: diffuse bilateral alveolar consolidation and ground glass changes	*T. pulmonis* diagnosed with 16S sequencing.	Empiric augmentin and azithromycin. Switched to 2 weeks ciprofloxacin.
Romano et al, 2011 (*7*)	70M with septic pulmonary emboli	Lung carcinoma, long-term central venous catheter, chemotherapy	Fever, Chills, dyspnoea and chest pain	CT: Bilateral pulmonary nodules	*T. tyrosinosolvens* by 16S rRNA sequencing	Levofloxacin for 6 weeks
Yang et al, 2017 (*8*)	24 F with pneumonia	None	20 d of fever and cough	CT: Small cavitary lesion and consolidation	*Tsukamurella* by 16S rRNA sequencing	19 d of linezolid
Sandhu et al, 2022 (*9*)	77M with atypical pneumonia	Asthma, lobectomy	Chronic cough, acute weakness, fevers, chills, night sweats.	CT: multiple regions of nodular parenchymal opacities.	*Tsukamurella*, unclear diagnostic method	Co-trimoxazole for 4 mo.
Liu et al, 2022 (*10*)	72M with Tsukamurella pneumonia misdiagnosed as TB	Daily dexamethasone, recurrent pneumonia	Productive cough and chest tightness	CT: L pyopneumothorax and RUL cavity	*T. tyrosinosolvens* identified using mass spectrometry post-mortem	Initial HRZE, then meropenem and linezolid

First author (year)	Details	Risk factors	Clinical presentation	Radiology	Diagnostic method and species	Treatment
Kuge et al, 2022 (*11*)	82F with Tsukamurella bronchiectasis misdiagnosed as NTM	Previous M. chelonae respiratory infection	Productive cough and hemoptysis	CT: Centrilobular nodules and bronchiectasis	*T. toyonakaense* identified using Multi Locus Sequence Typing and whole genome sequencing	20 y erythromycin. Patient declined clarithromycin with ethambutol therapy.
Gotoh et al 2021 (*12*)	44F with septic pulmonary embolism	Central venous catheter and cisplatin chemotherapy	High fever during chemotherapy and radiotherapy	CT: Bilateral pulmonary nodules consistent with septic pulmonary emboli	*T. inchonensis* by *groEL* sequencing	Imipenem and cilastatin for 4 weeks
Swier et al 2022 (*13*)	73F with exacerbation of bronchiectasis	Bronchiectasis	Worsening cough and sputum production with weight loss	N/A	*Tsukamurella sp.* by 16S rRNA sequencing	N/A
Manek et al, 2019 (*14*)	42M with recurrent pleural effusion	None	Chest pain and shortness of breath	CXR: R sided pleural effusion	*T. pulmonis* (diagnostic method not described)	Initial HRZE. Then 2 mo levofloxacin and rifabutin for 3–6 mo.
Candelario et al, 2019 (*15*)	80F with frequent exacerbations of COPD	COPD	Worsening productive cough	CT chest: emphysematous changes with multiple nodules	*T. pulmonis* diagnosed using 16S sequencing	2 mo azithromycin therapy
Rao et al, 2023 (*16*)	68F	Pulmonary fibrosis, past TB	Shortness of breath, fatigue and weight loss for 6 mo	CT chest- multifocal pneumonia with cavitary lesions	*Tsukamurella sp.* by chromatography	Levofloxacin & rifampin for 3 mo

References

1. Ménard A, Degrange S, Peuchant O, Nguyen TD, Dromer C, Maugein J. *Tsukamurella tyrosinosolvens*—an unusual case report of bacteremic pneumonia after lung transplantation. Ann Clin Microbiol Antimicrob. 2009;8:30. PubMed https://doi.org/10.1186/1476-0711-8-30

2. Maalouf R, Mierau SB, Moore TA, Kaul A. First case report of community-acquired pneumonia due to *Tsukamurella pulmonis.* Ann Intern Med. 2009;150:147–8. PubMed https://doi.org/10.7326/0003-4819-150-2-200901200-00022

3. Perez VA, Swigris J, Ruoss SJ. Coexistence of primary adenocarcinoma of the lung and *Tsukamurella* infection: a case report and review of the literature. J Med Case Rep. 2008;2:207. PubMed https://doi.org/10.1186/1752-1947-2-207

4. Alcaide ML, Espinoza L, Abbo L. Cavitary pneumonia secondary to *Tsukamurella* in an AIDS patient. First case and a review of the literature. J Infect. 2004;49:17–9. PubMed https://doi.org/10.1016/S0163-4453(03)00139-7

5. Mehta YB, Goswami R, Bhanot N, Mehta Z, Simonelli P. *Tsukamurella* infection: a rare cause of community-acquired pneumonia. Am J Med Sci. 2011;341:500–3. PubMed https://doi.org/10.1097/MAJ.0b013e3182129d02

6. Inchingolo R, Nardi I, Chiappini F, Macis G, Ardito F, Sali M, et al. First case of *Tsukamurella pulmonis* infection in an immunocompetent patient. Respir Med CME. 2010;3:23–5. https://doi.org/10.1016/j.rmedc.2009.02.004

7. Romano L, Spanu T, Calista F, Zappacosta B, Mignogna S, Sali M, et al. *Tsukamurella tyrosinosolvens* and *Rhizobium radiobacter* sepsis presenting with septic pulmonary emboli. Clin Microbiol Infect. 2011;17:1049–52. PubMed https://doi.org/10.1111/j.1469-0691.2010.03396.x

8. Yang L, Cao Y, Dan Z, Wang Z, Wang X. Community-acquired *Tsukamurella* pneumonia in a young immunocompetent adult: a case misdiagnosed as pulmonary tuberculosis and literature review. Postgrad Med. 2017;129:563–6. PubMed https://doi.org/10.1080/00325481.2017.1344513

9. Sandhu JE, Ariyaratnam J. D Trandafirescu T. *Nocardia Tsukamurella*: an atypical pneumonia. Chest. 2022;162(Supplement):A561. https://doi.org/10.1016/j.chest.2022.08.435

10. Liu C-Y, Lai C-C, Lee M-R, Lee YC, Huang YT, Liao CH, et al. Clinical characteristics of infections caused by *Tsukamurella* spp. and antimicrobial susceptibilities of the isolates. Int J Antimicrob Agents. 2011;38:534–7. PubMed https://doi.org/10.1016/j.ijantimicag.2011.07.018

11. Kuge T, Fukushima K, Matsumoto Y, Saito H, Abe Y, Akiba E, et al. Chronic pulmonary disease caused by *Tsukamurella toyonakaense.* Emerg Infect Dis. 2022;28:1437–41. PubMed https://doi.org/10.3201/eid2807.212320

12. Gotoh K, Mayura IPB, Hagiya H, Obata K, Ogawa T, Iio K, et al. Septic pulmonary emboli caused by *Tsukamurella inchonensis*: A case report. J Infect Chemother. 2021;27:369–72. PubMed https://doi.org/10.1016/j.jiac.2020.09.024

13. Swier R, Jakharia KK. Acid-fast bacteria in bronchiectasis: if the glass slipper does not fit, non-TB mycobacteria, consider *Tsukamurella*. Chest. 2022;162:A573. https://doi.org/10.1016/j.chest.2022.08.444

14. Manek G, Shah N, Krishnan AM, Anthony P. A case of *Tsukamurella pulmonis* masquerading as pleural TB in an immunocompetent patient. Chest. 2019;156(Supplement):A252. https://doi.org/10.1016/j.chest.2019.08.302

15. Akkineni S, Calderon Candelario RA, Mirsaeidi M. Acute COPD exacerbation associated with a rare pathogen: a case of *Tsukamurella pulmonis.* Am J Respir Crit Care Med. 2019;199:A6448. https://doi.org/10.1164/ajrccm-conference.2019.199.1_MeetingAbstracts.A6448

16. Rao S, Paz M, Nugent K. *Tsukamurella* and *Mycobacterium tuberculosis* pneumonia co-infection. Am J Med Sci. 2023;365:S163-S. https://doi.org/10.1016/S0002-9629(23)00312-9
